# Supplementary material for: Oncolytic H-1 Parvovirus Hijacks Galectin-1 to Enter Cancer Cells
Source: Viruses. 2022 May 11;14(5):1018. doi: 10.3390/v14051018 (PMC9146882; doi:10.3390/v14051018)
Supplement: Supplementary file 1 [file viruses-14-01018-s001.zip › viruses-1703502-supplementary.pdf]

Ferreira et al. 2022

Oncolytic H-1 Parvovirus Hijacks Galectin-1 to Enter Cancer Cells

Supplementary Figures

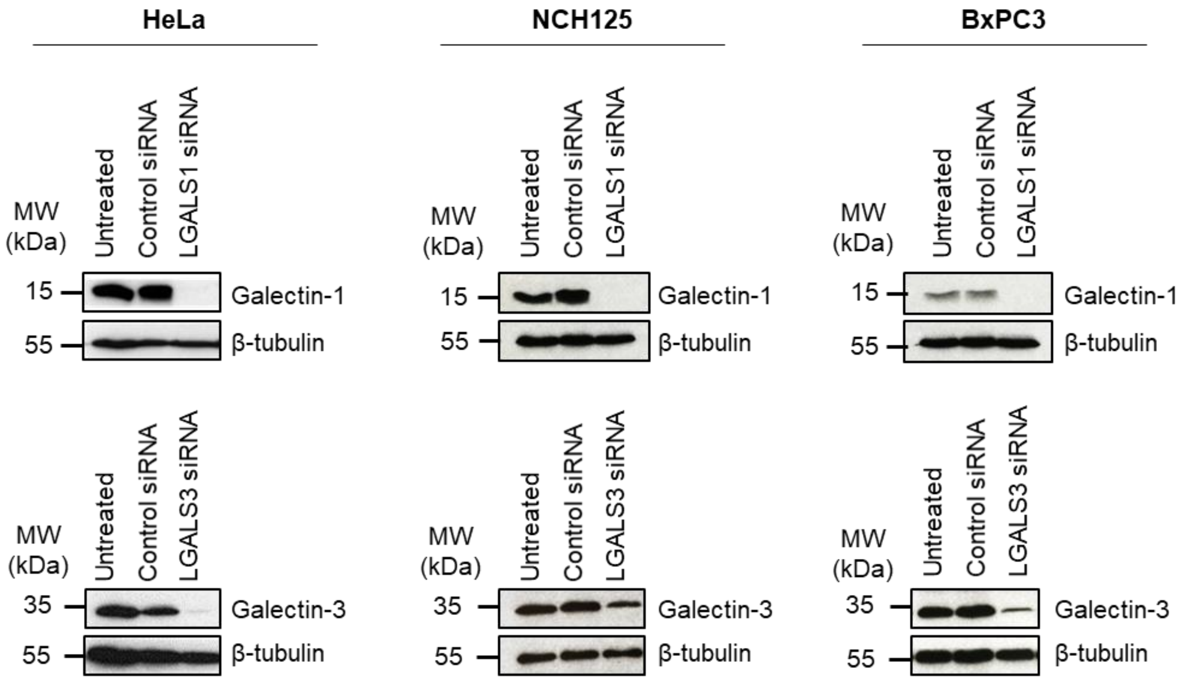

Figure S1. LGALS1 and LGALS3 knockdown in HeLa, NCH125 and BxPC3 cell lines. HeLa, NCH125 and BxPC3 cells were transfected with siRNAs targeting *LGALS1* or *LGALS3* or with a scrambled siRNA. At 48 hours post-transfection, the protein levels of Gal-1 and Gal-3 on cell lysates were analysed by Western blotting. Beta-tubulin was used as a loading control.

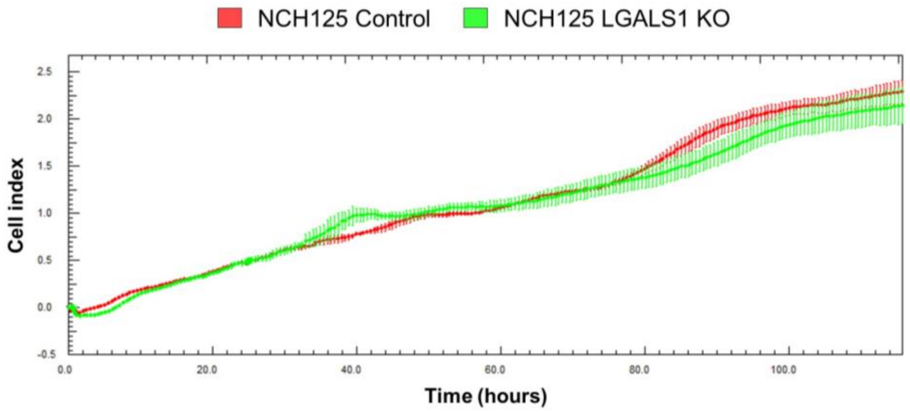

Figure S2. Cell proliferation of NCH125 Control *versus* NCH125 LGALS1 KO. NCH125 Control and LGALS1 KO cells were seeded in a 96-well E-plate and grown for five days. Cell proliferation was monitored with the xCELLigence System. Curves represent the mean Cell Index ± standard deviation (n=3).

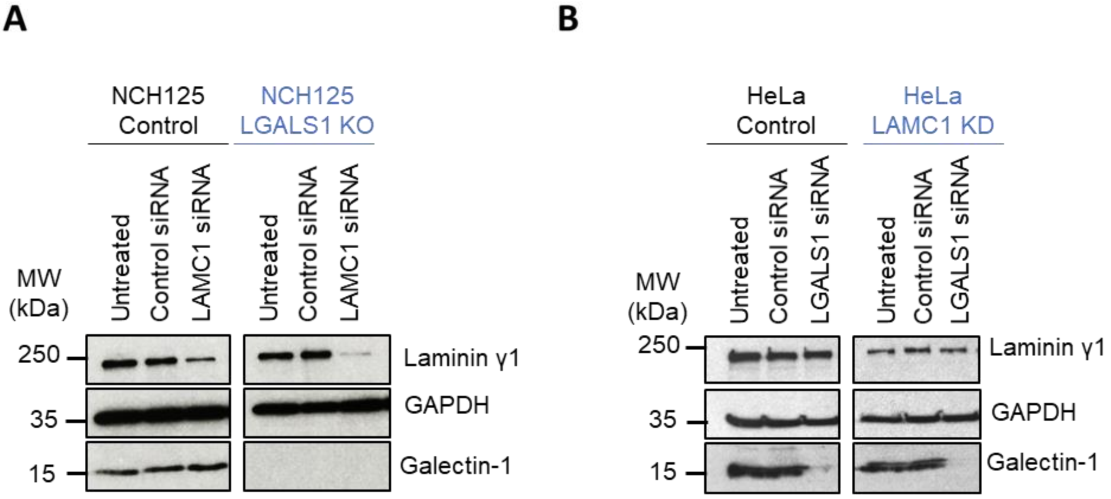

Figure S3. Depletion of both *LAMC1* and *LGALS1* in NCH125 and HeLa cells. **(A)** NCH125 Control and LGALS1 KO cells were transfected with a siRNA targeting *LAMC1* or a negative control siRNA. **(B)** HeLa Control and HeLa LAMC1 KD cells were transfected with a siRNA targeting *LGALS1* or a negative control siRNA.At 48 hours post-transfection, the protein levels of laminin γ1 and Gal-1 on cell lysates were analysed by Western blotting. GAPDH was used as a loading control.

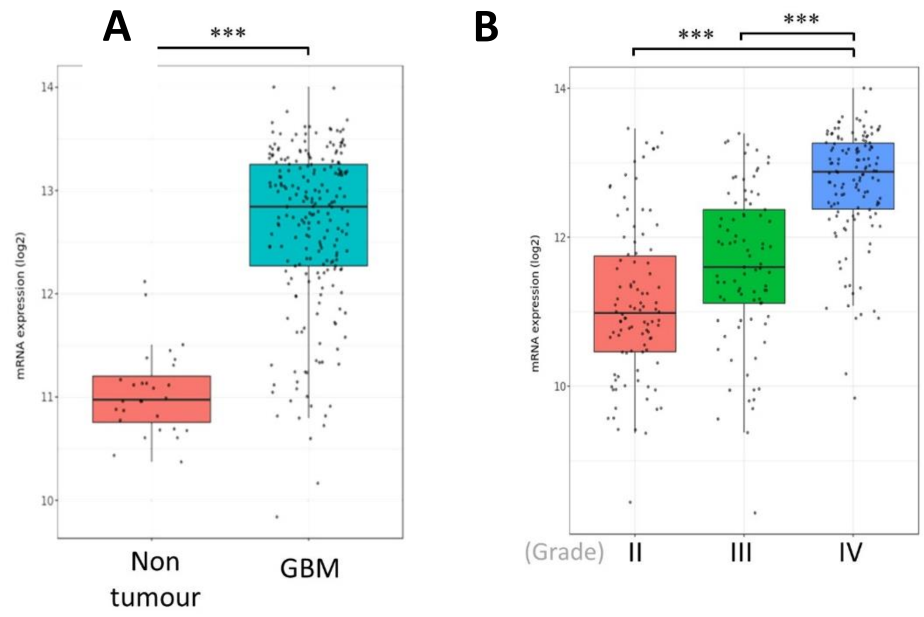

Figure S4. *LGALS1* overexpression in high-grade GBM. Analysis of *LGALS1* expression retrieved from the REMBRANDT dataset using the GlioVis portal. **(A)** Comparison based on histology, namely non-tumour *versus* glioblastoma multiforme (GBM). **(B)** Comparison based on WHO grade II, III and IV gliomas. Statistical significance was assessed by Tukey's honestly significant difference test (****$p<0.001$*).

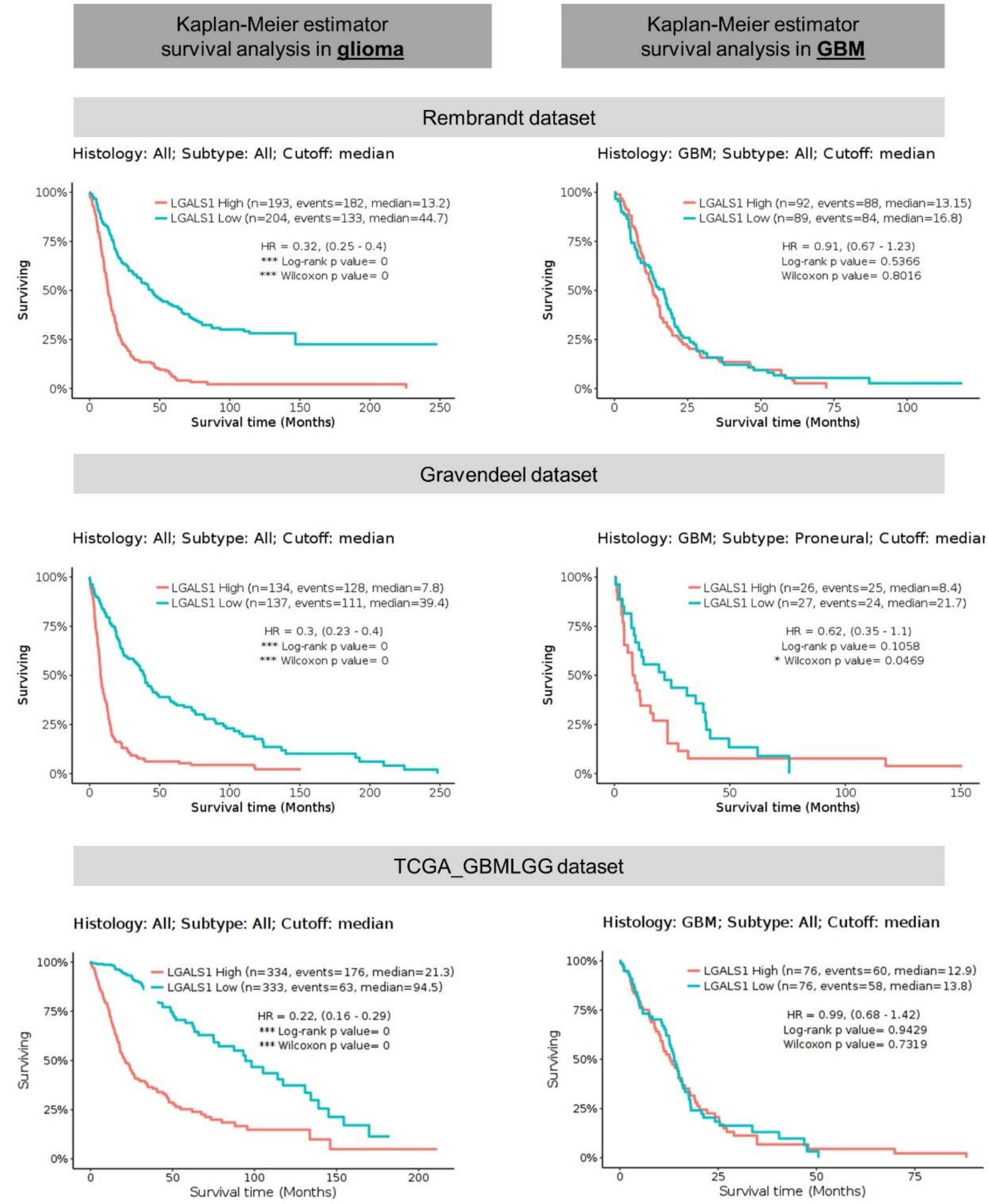

Figure S5. Higher levels of *LGALS1* are associated with poor prognosis in glioma. Kaplan-Meier survival curves for patients with glioma or glioblastoma (GBM) are divided into two groups based on *LGALS1* expression. Analysis was retrieved from the Rembrandt, Gravendeel and TCGA_GBMLGG datasets using the GlioVis portal. The number of patients in each group and the log-rank *p* value are indicated. HR: hazard ration.

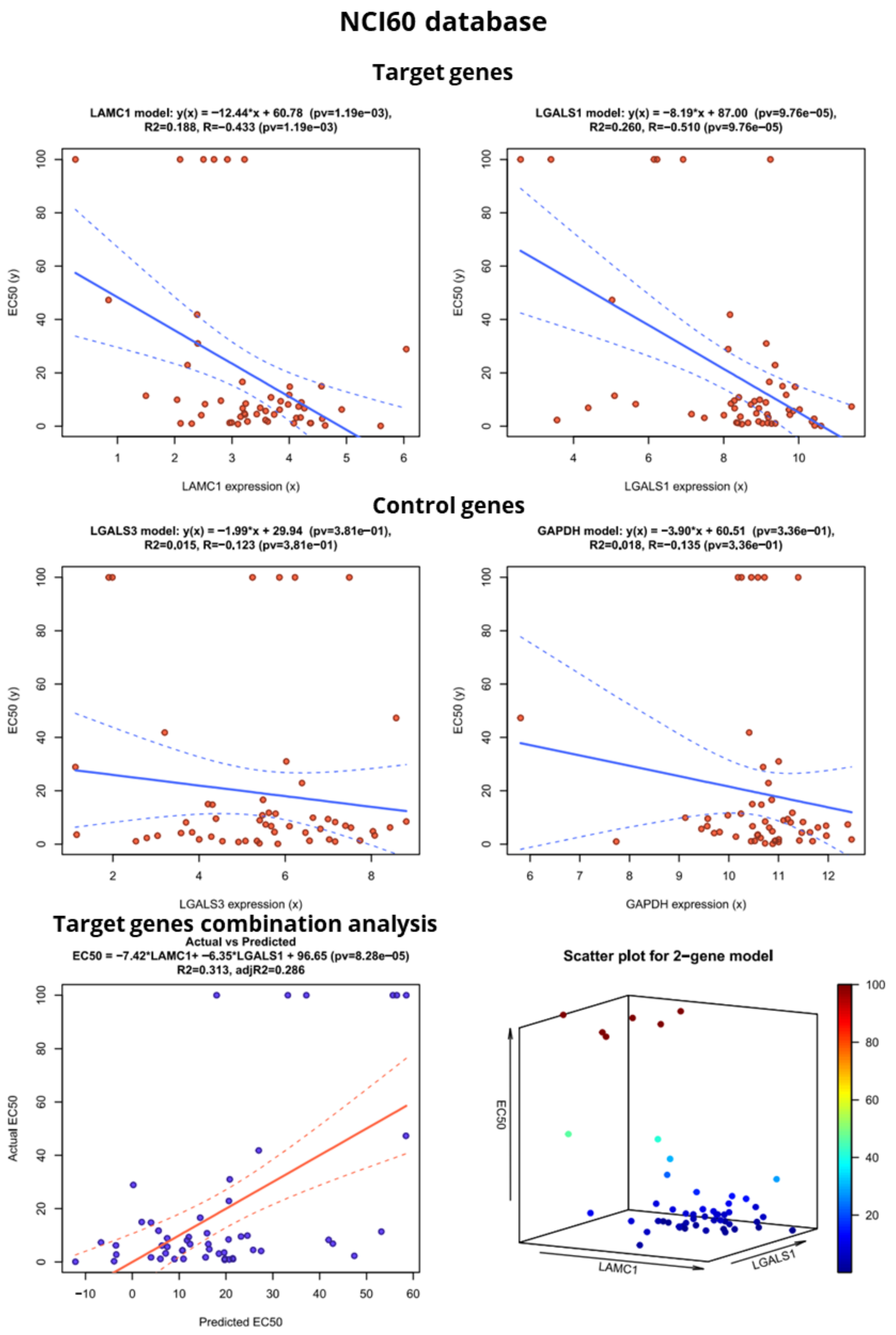

Figure S6. NCI60 database retrieved *LGALS1* and *LAMC1* expression levels showed anti-correlation with EC50 values and a slight increase in the ability to predict susceptibility to H-1PV induced oncotoxicity. Top plots show single-gene scatter plots with a linear model (dotted line – 95% C.I.) for the genes of interest. Middle plots – same for the control genes. Bottom left plot – comparison of the predictions by the two-gene model with experimental EC50 values. Bottom right – scatter plot in 3 dimensions (genes of interest and EC50).

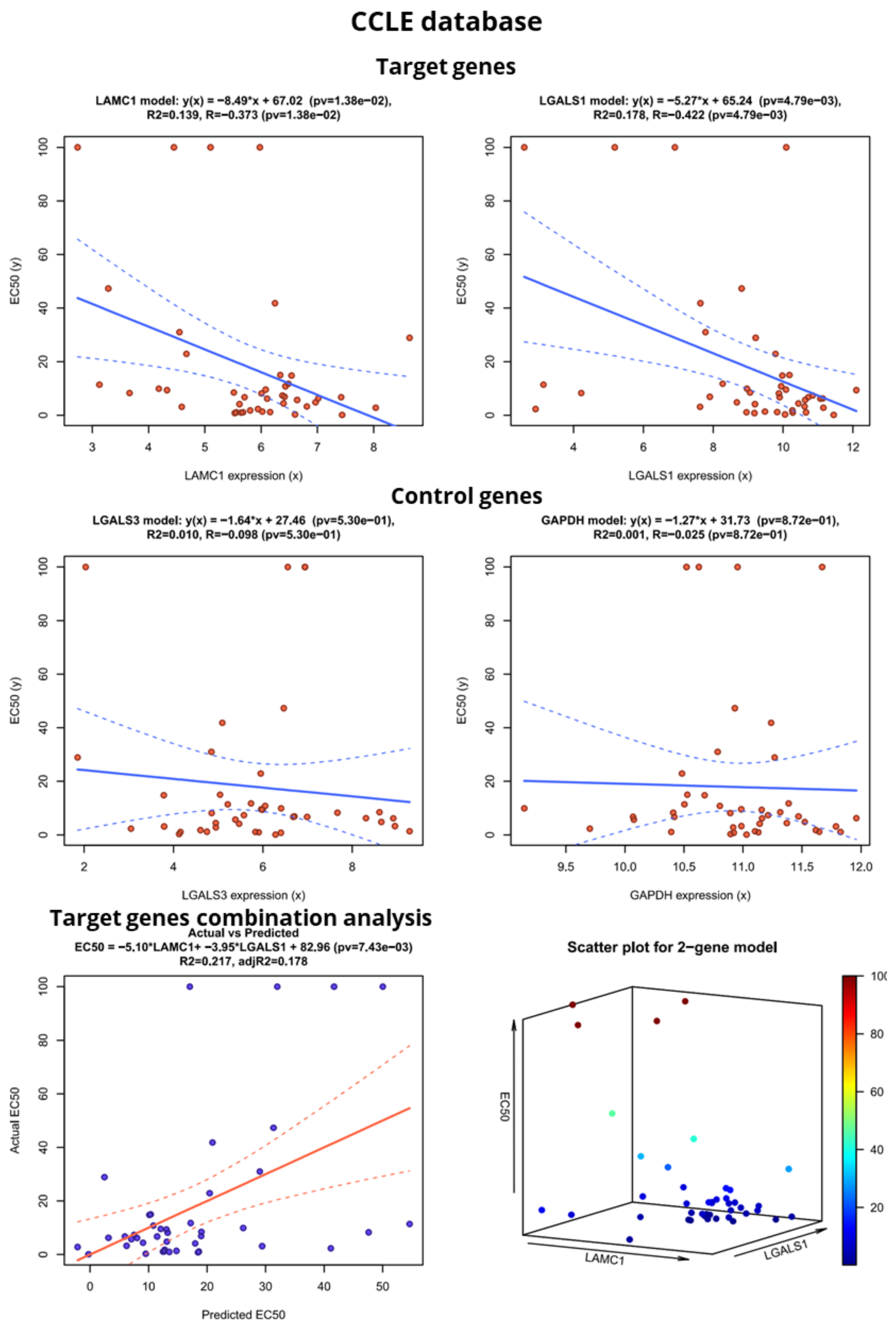

Figure S7. CCLE database retrieved *LGALS1* and *LAMC1* expression levels showed anti-correlation with EC50 values and shows a slight increase in the ability to predict susceptibility to H-1PV induced oncotoxicity. Top plots show single-gene scatter plots with a linear model (dotted line – 95% C.I.) for the genes of interest. Middle plots – same for the control genes. Bottom left plot – comparison of the predictions by the two-gene model with experimental EC50 values. Bottom right – scatter plot in 3 dimensions (genes of interest and EC50).
